# Two-Photon Endoscopy: State of the Art and Perspectives

**DOI:** 10.1007/s11307-021-01665-2

**Published:** 2021-11-15

**Authors:** Vytautas Kučikas, Maximilian P. Werner, Thomas Schmitz-Rode, Frédéric Louradour, Marc A. M. J. van Zandvoort

**Affiliations:** 1 Institute for Molecular Cardiovascular Research (IMCAR), RWTH Aachen University, Aachen, Germany; 2 XLIM Research Institute, Limoges University, CNRS, Limoges, France; 3 Department of Biohybrid and Medical Textiles (BioTex), RWTH Aachen University, Aachen, Germany; 4 Institute for Cardiovascular Diseases CARIM, Department of Molecular Cell Biology, Maastricht University, Maastricht, Netherlands

**Keywords:** Multiphoton endoscopy, Nonlinear endoscopy, In vivo imaging, Label free, Kidney imaging, Colon imaging

## Abstract

In recent years, the demand for non-destructive deep-tissue imaging modalities has led to interest in multiphoton endoscopy. In contrast to bench top systems, multiphoton endoscopy enables subcellular resolution imaging in areas not reachable before. Several groups have recently presented their development towards the goal of producing user friendly plug and play system, which could be used in biological research and, potentially, clinical applications. We first present the technological challenges, prerequisites, and solutions in two-photon endoscopic systems. Secondly, we focus on the applications already found in literature. These applications mostly serve as a quality check of the built system, but do not answer a specific biomedical research question. Therefore, in the last part, we will describe our vision on the enormous potential applicability of adult two-photon endoscopic systems in biological and clinical research. We will thus bring forward the concept that two-photon endoscopy is a sine qua non in bringing this technique to the forefront in clinical applications.

## Introduction

Fluorescence is one of the most frequently used contrast methods in optical microscopy. It arises from excited fluorophores emitting visible light during their return to the ground state. The different microscopy techniques branch in the way of fluorophore excitation and fluorescence signal collection. In the linear microscopy, near ultraviolet or visible spectrum (UV–VIS, 320–700 nm) emitting LEDs (in wide field microscopy, WFM) or lasers (in confocal laser scanning microscopy, CLSM) are used to excite fluorophores. This excitation takes place in the illuminated volume, either in the whole tissue (in WFM) or in the conical volume of the laser beam focusing path (in CLSM). Such large excitation volume results in a high amount of phototoxicity and photobleaching. Furthermore, the fluorescence signal is collected from the whole illuminated volume, resulting in signal from out-of-focus planes. In CLSM, spatial filtering is used to distinguish the fluorescence signal of the focused position from that coming from out-of-focal planes. In this way, the image is built up by scanning the laser beam across the whole plane of imaging. This is quite effective in the surface regions (not more than 50 µm in depth), allowing to obtain 3D information by making stacks of images. However, imaging deeper in the tissue the signal strength reduces rapidly, because emitted photons get scattered by the shallower layer of the tissue and then filtered out by the pinhole as noise. Also, due to this scattering, light originating from outside of the focal spot might be accepted by the pinhole.

**Fig. 1.**
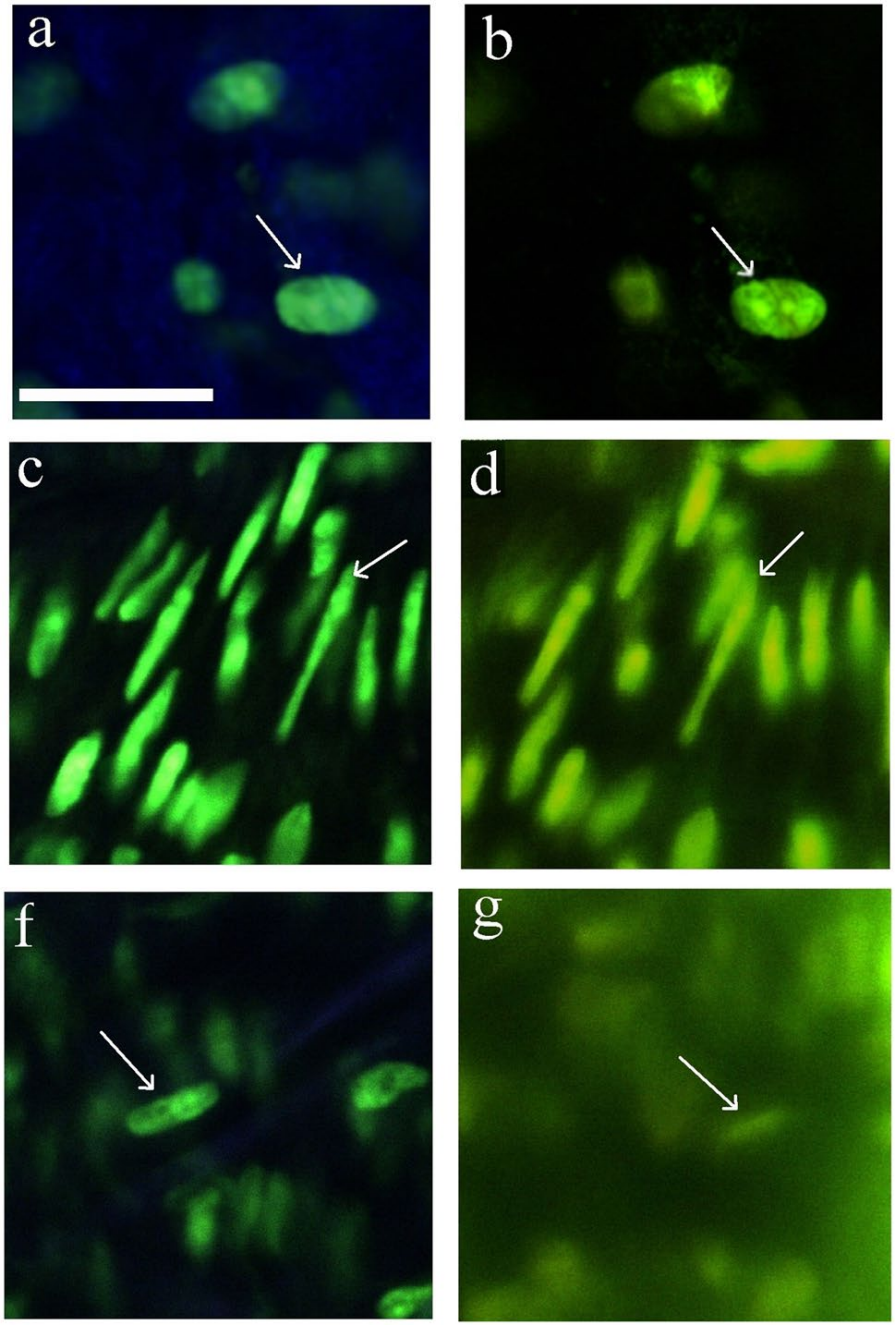
Comparison of two-photon and confocal microscopies. TPLSM (**a,c,e**) and CLSM (**b,d,g**) images of mounted carotid artery, stained for cell nuclei with Syto13. The quality of images is compared in different depths of the sample: at the surface (**a,b**); 40 µm below (**c,d**), and 80 µm below (**f,g**) the surface [1].

Two-photon laser scanning microscopy (TPLSM) is similar to CLSM in that a laser beam is scanned through the tissue volume, collecting signal from every voxel separately. However, the wavelength used for the excitation is double (and thus the energy of the excitation-involved photons is half) compared to that in CLSM. To enable fluorophore excitation with two photons, these photons need to appear in simultaneous atomic proximity, i.e., at the same position and within a very short time frame of femtoseconds. This combination of spatial and temporal prerequisites results in several advantages, as well as in increased complexity of the system: (1) The excitation only takes place in the region where light intensity is concentrated. This is a key feature of two-photon microscopy, as it leads to the localization of excitation to the focal spot of the laser beam, reducing noise, potential photodamage, and omitting requirement for pinhole filtering. Indeed, this means that even in the presence of multiply-scattered signal, the origin of fluorescence light is always known to be the focal spot. Scattering thus has less influence on signal-to-noise ratio [1]. On the other hand, only femtosecond pulses can achieve such intensities, leading to complex and expensive laser systems and specific pulse handling, which will be discussed in the later sections. (2) The wavelengths of the excitation pulses are in the near infrared region (NIR), thus having better penetration depth and reduced scattering in the tissues. This gives the possibility to image tissues up to 0.5–1 mm under the surface. A comparison between confocal and TPLSM in that respect is given in Fig. 1. One specific example for better penetration is blood, which has much lower absorbance in NIR compared with visible light. On the other hand, since diffraction-limited resolution is proportionally dependent on wavelength, at shallow depths where scattering is negligible, CLSM reaches better resolution than TPLSM with the same focusing parameters. (3) TPLSM can be used for optical second harmonic imaging. Non-centrosymmetric biocrystalline structures, such as collagen, microtubules, or muscle myosin, can generate second harmonic signals. Second harmonic generation (SHG) does not involve exited states of the molecules, effectively negating photobleaching and phototoxicity. Furthermore, the wavelength of emission of the second harmonic signal is exactly half that of the excitation, making it easily distinguishable from autofluorescence, effectively adding an additional information channel to the image.

In the last two decades, TPLSM has become a valuable tool for high-resolution imaging of morphology, physiology, and cell-to-cell interactions in live tissues and animals. Its potential to image tissue at depths unreachable by linear optical techniques (such as CLSM) with reduced photodamage [2–4] led to a huge interest in applying TPLSM to *in vivo* animal imaging. However, conventional bench top multiphoton microscopes often have limitations in tissue accessibility, and therefore a large range of *in vivo* applications are not feasible. Although bench top multiphoton microscopes are used for human applications in dermatology [5], internal organs are not accessible by such technology.

Although multiphoton microscopy has been around for over 20 years now and is the method of choice for deep-tissue imaging, in the past five years, new developments from a technological point of view have been limited, and focus has been on broadening biomedical applications, mostly in the fields of cardiovascular, oncological, brain, retinal, kidney, and liver. Clinical applications so far have been seldom.

A new and rather unexplored potential of two-photon microscopy lies in endoscopy, since this would solve the problem of the bulky, expensive standard two-photon microscopy and would furthermore allow, in the future, intravital and clinical patient imaging. Examplatory, we mention the enormous potential of two-photon endoscopy in clinical research on epilepsy, placenta (pre-eclampsia), bladder (oncology, Alzheimer), cardiovascular (heart valves, atherosclerosis), and oncological (skin, deeper tumors, photodynamic therapy). Instead of having to take out tissue of, e.g., a tumor, do histology, and come up with a result 10 days later (which indeed means ten nerve-wrecking days for the patient), this would allow instantaneous next-to-the-bed diagnosis.

**Fig. 2.**
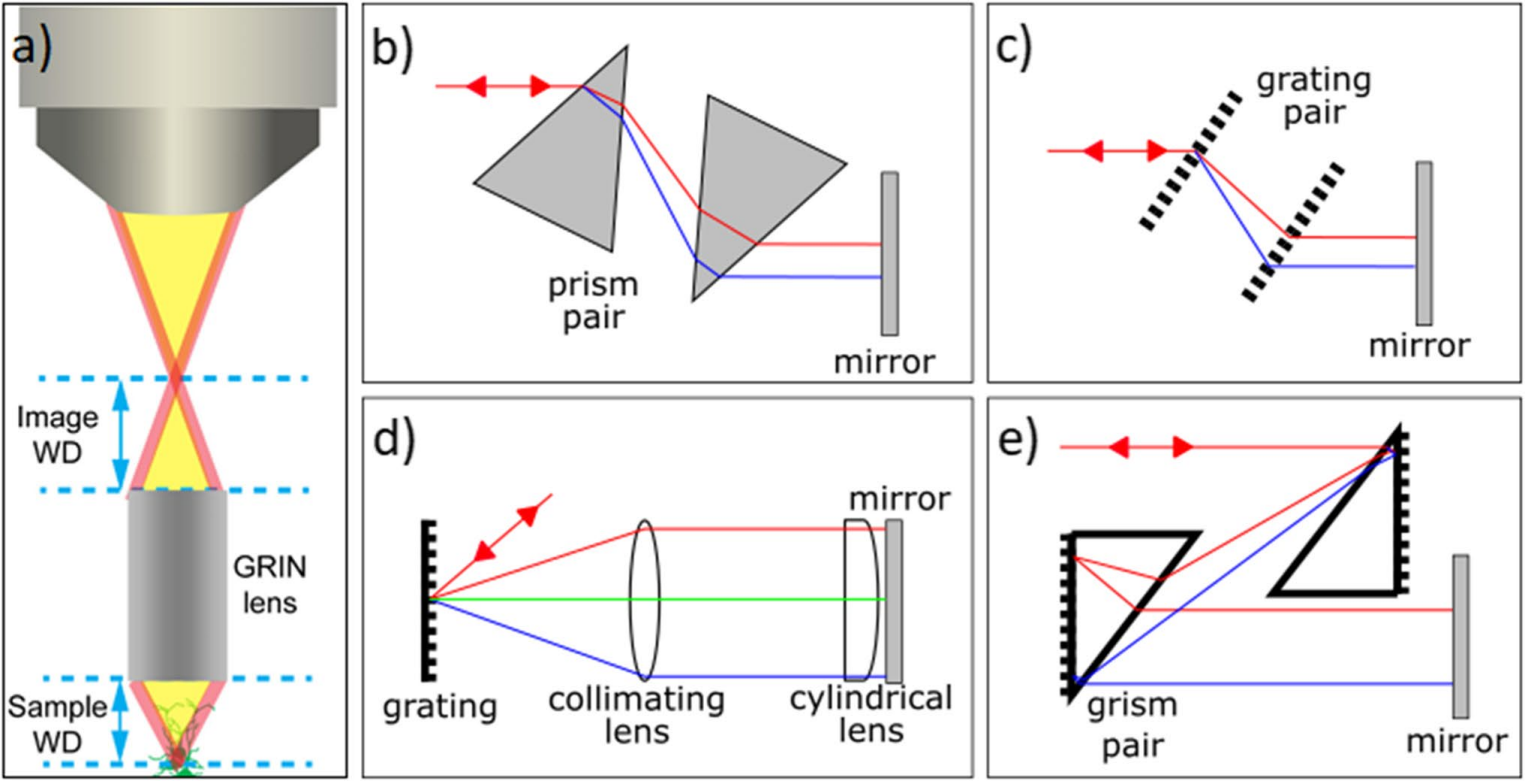
Principal drawing of GRIN based needle endoscope [9] (**a**) and different pulse stretching (pre-compensation) mechanisms: **b** prism pair, usually implemented inside laser resonator [13]; **c** diffraction gratings stretcher [14]; **d** grating plates combined with cylindrical lens [15]; **e** diffraction gratings and prisms combination (grism), addressing second and third order dispersions [16].

Earliest attempts to construct two-photon endoscopic systems were based on the bulky bench top setups, paired with a long (tens of millimeters) and thin (1–2 mm, therefore resembling a needle) imaging lens, used in front of the microscope objective. It translates the focal spot to an intermediate plane, which is observed by the main objective (Fig. 2a). Usually, GRIN (gradient index) lenses are used in this approach. Refraction in GRIN lenses is achieved by a chemical composition gradient, rather than by their form, potentially allowing arbitrary dimensions. However, an important problem of GRIN lenses is strong chromatic aberration. Nevertheless, long GRIN lenses give some flexibility in reaching tissues in, e.g., *in vivo* colonoscopy, kidney, liver [6–8], or brain imaging in mice *in vivo* [9] as well as ex vivo [10], and ex vivo human tissues in pursuit to lay foundations for *in vivo* experiments [11]. Some companies started to produce these so-called needle microscopes with true lenses instead of GRIN, solving chromaticity problems [12]. However, their endoscopic abilities are rather limited. Therefore, they will not be discussed further in this paper.

We will focus on fiber-based endoscopic systems currently being developed, using a miniaturized probe that is connected to a laser excitation source and to an analyzing system via a flexible optical fiber. The use of label-free fluorescence in living humans is desirable, although biocompatible exogenous markers are clinically accepted, e.g., indigocarmine or methylene blue. The development of endoscopic systems experiences some common difficulties in both confocal and two-photon microscope systems. These include the development of scanning systems, probe miniaturization, and focusing systems.

Confocal endoscopy has already reached commercialization, with the systems that are plug and play and understandable for users without background in engineering and physics [17]. The same goal is set for multiphoton endoscopic systems. With increasing theoretical and practical knowledge, a huge step towards this goal has been made in the recent decades. The progresses in short pulse laser technology, resulting in fiber lasers with low cost, less complexity, and fragility, make multiphoton-based endoscopic technology ready to be applied in biomedical research. We are convinced that, in the near future, it will lead to a breakthrough in clinical applications, e.g., imaging of internal organs. Not only does it provide subcellular resolution in organs and tissues that could not be reached before, it also enables entirely new possibilities.

## Basics of Fiber-Based TPLSE Systems

### The Underlying Concept: Group Velocity Dispersion and Kerr Effect

Optical fibers have been proved to be a valuable tool in imaging systems, due to their flexibility. Their foremost application in microscopy is the delivery of excitation light and collection of fluorescence light to and from an arbitrary location, making microscopy of internal organs feasible even in *in vivo* applications. However, their incorporation in two-photon microscopy causes additional challenges, due to some fundamental effects of nonlinear optics. The full mathematical explanation and analysis of arising problems can be found in the literature [18]. In the following, we will shortly review the most important of them and the problems they cause, since the engineering solutions of TPLSE components are highly determined by them.

The product of laser pulse duration and spectral bandwidth has a theoretical minimum, called the Fourier transform limit. E.g. 850 nm central wavelength and 100 fs duration Gaussian pulse (common for excitation in TPLSM) has inevitably broader spectrum than 10 nm. Unfortunately, most of the fiber cores are composed of dispersive media, meaning that velocities in the whole group of different wavelengths slightly differ (group velocity dispersion, GVD), and therefore at the end of the transfer, duration of the pulse is significantly increased. Every dispersive media has its specific chromatic dispersion pattern, but generally there are two different types of regions. In the normal dispersion region, longer waves travel faster and as a consequence the pulse will be up-chirped (longer wavelengths in the front and shorter in the tail of the pulse). In the anomalous dispersion region, shorter waves travel faster. As the consequence the pulse will be down-chirped (shorter wavelengths in the front and longer in the tail). Therefore, the ideal pulse, i.e., the shortest in time, is un-chirped since arrival time of wavelengths overlaps. This can be achieved by combining a normal (in TPLSE usually the endoscopic fiber) and an anomalous (in TPLSE usually a pre-compensation stretcher) dispersive media, in that way compensating dispersion effects. In general, chromatic dispersion is characterized by several orders of dispersion: 2nd order (SOD or GVD), 3rd order (TOD), and 4th order (FOD). In TPLSE, usually bandwidth is below 30 nm; therefore, TOD and FOD can be neglected, and GVD compensation is relatively easy. However, broader bandwidth is used in some compensation mechanisms, where TOD compensation must be addressed as well, while maintaining FOD at minimum. At the current state of the art, compensation of all—SOD, TOD, and FOD—is not feasible.

Another important aspect to consider is that very intense light induces transient (i.e., short-lasting) augmentation of optical density of the media. This reduces the speed of light propagating in the media, the so-called optical Kerr effect. As a consequence, at the slopes of the pulse (where intensity changes fastest), this causes either expansion of the light carrier wave (in the pulse’s front) or compression (in the pulse’s tail). This effect slightly alters the instantaneous frequency. Such modulation is called self-phase modulation (SPM). In most cases (i.e., when the pulse is un-chirped or up-chirped), SPM results in creation of new wavelengths, making spectrum of the pulse broader and thus more sensitive to GVD. In contrast, down-chirped pulses undergo the inverse effect: Their spectrum is narrowed by SPM, leading to longer pulse duration. Conclusively, GVD and SPM must be addressed very carefully, as their combined effect in the endoscopic fiber might prolong femtosecond pulse to several tens of picoseconds, reducing pulse intensity and making the probability of two-photon absorption negligibly small.

It is obvious from the above that delivery of femtosecond pulses through a fiber (as needed for fiber-optical TPLSE) is not an easy goal. Therefore, below, we will describe the required characteristics of the main components of fiber-based TPLSE systems. We will discuss the following topics:

1. Mechanism of pre-compensation. Since the use of a distal probe requires severe miniaturization, excitation pulses have to be pre-compensated prior being injected into the fiber: transformed in such a way that dispersion and nonlinear effects of the endoscopic fiber would reconstruct them into shortest pulses possible. Therefore, a so-called pulse stretcher is inserted before the endoscopic fiber, while the fiber plays the role of a pulse compressor.
2. Selection of fiber type. An endoscopic fiber has to fulfill two important requirements. Firstly, it has to be capable to deliver excitation pulses which have to remain short (e.g., 100 fs). Secondly, it has to be capable to collect a significant amount of VIS emission signal from the sample. In this part, we will show that while these two requirements seem to be contradictive, solutions requiring specific fiber characteristics do exist. To that end we will discuss the various types of fibers.
3. Methods of focusing. After the excitation IR light has left the fiber, it has to be focused on the sample. This focusing system should be localized in a small probe (lower than 3 mm outer diameter is highly desired) to allow the application for internal organs. Thus, miniaturization of focusing systems is an important task in fiber-based TPLSE.
4. Methods of scanning. Like in any confocal or MPLSM microscope, the focal spot has to be scanned over the sample to achieve an image of a single plane. Scan speed is a limiting factor in real-time applications. Bench top microscopes use galvanometric scanners reaching frame rates of 30 fps. However, these bulky systems cannot be minimized to acceptable dimensions for the endoscopic probe.

In the following, we describe the current (limited) applications of fiber-based TPLSE and discuss a future path towards wider use of these very promising systems.

### Mechanism of Pre-compensation

A simple solution for dispersion pre-compensation is the dispersive prism pair (Fig. 2b). Dispersion can be adjusted varying the insertion of one or both prisms into the beam path. However, achievable group delay dispersion (GDD) is very limited, consequently limiting endoscopic fiber length [13].

The most widely used anomalous pulse stretcher is composed of two diffraction gratings (Fig. 2c) [19–24]. The antiparallel grating pair is positioned in the optical path, forcing diffracted spectral components to travel different paths in air and get recomposed afterwards using a folding mirror imposing back and forth propagation inside the stretcher. Diffraction gratings produce a much greater angular dispersion compared to prisms; therefore, the achievable GDD is increased. The amount of stretching can be adjusted by changing the distance between the two gratings and the angle of incidence onto the first grating. The main drawback, however, is that such stretcher has large TOD that cannot be tuned.

An interesting approach is to use a single diffraction grating combined with a cylindrical lens (Fig. 2d). The spectral components are angularly separated by the grating, collimated by a spherical lens, and directed to the cylindrical lens (convex only along one transverse axis). In this scheme, the dispersion compensation can be tuned by rotating the cylindrical lens around the optical axis, effectively changing the distances travelled by the different spectral components inside the cylindrical lens [15]. This setup requires that the cylindrical lens has negligible effect on the direction of the spectral components; therefore, only very long focal distance cylindrical lenses can be used. Since the effectiveness of such stretcher is reversely dependent on the focal distance of the cylindrical lens, the achievable GDD is a tradeoff of spatial aberration. Nevertheless, effective systems containing such pre-compensation mechanism were reported [25]. 

The drawback of all of the above-mentioned methods is that pulse stretching is purely linear, i.e., without change in the (width of) spectrum. While this can be perfect for low intensity pulses, highly intense pulses experience severe spectral narrowing due to SPM during compression in the endoscopic fiber. Such narrowed spectrum implies a longer duration pulse due to the Fourier transform limit. Consequently, even perfectly pre-compensated pulses become much longer at the fiber output. To overcome this, multiple steps pre-compensation mechanisms have been reported [16, 26–29]. The initial pulse is focused to a polarization maintaining single-mode fiber acting as a nonlinear element. In this additional fiber, pulse spectrum is widely broadened by SPM. Then, in the pulse stretcher, the pulse is affected linearly, and its spectral bandwidth is not modified. Therefore, the pulse at the entrance of the endoscopic fiber not only is down chirped, but also has a significantly broader spectrum. Even with the spectral narrowing in the endoscopic fiber, the spectrum stays wide enough to keep compressed pulses as short as initial, or even shorter. The only problem arising from this setup is that the pulse spectrum after the broadening becomes extremely wide, reaching over a 100 nm. With such wide bandwidth, the dispersion of the media becomes much more complex, and SOD compensation is not enough. While stretcher consisting of diffraction gratings was shown to be applicable in the systems where endoscope fiber is relatively short [30], it is shown that conventional grating-pair compressor cannot be designed with zero TOD [31]. For this reason, a more sophisticated stretcher was introduced, combined with diffraction gratings and prisms used in a very close assembly, creating *grism* elements [32, 33] (Fig. 2e). With such *grism* elements, SOD and TOD can be separately corrected, while keeping FOD at relatively low level. With 150 fs 820 nm central wavelength excitation pulses, it was shown that even sub-30 fs light pulses are achievable by such compensation system and 2.7-m-long endoscopic fibers [26]. While grating-based compensation could compete with reflective grism systems due to the higher throughput, recent developments in transmission-type *grism* make them even more efficient [34, 35].

In summary, pre-compensation is a challenging task in all femtosecond fiber systems, including two-photon endoscopy. Even though endoscopic systems with simpler mechanisms report successful two-photon imaging, complex and sensitive systems (as the *grism* stretcher) allow optimal delivery of femtosecond pulses, increasing signal-to-noise ratio and penetration depth. Consequently, lower thermal effects are induced in the specimen allowing higher signal collection without harming the tissue.

### Selection of the Fiber Type

#### Conventional Single-Mode and Multimode Fibers (Fig. 3a)

**Fig. 3.**
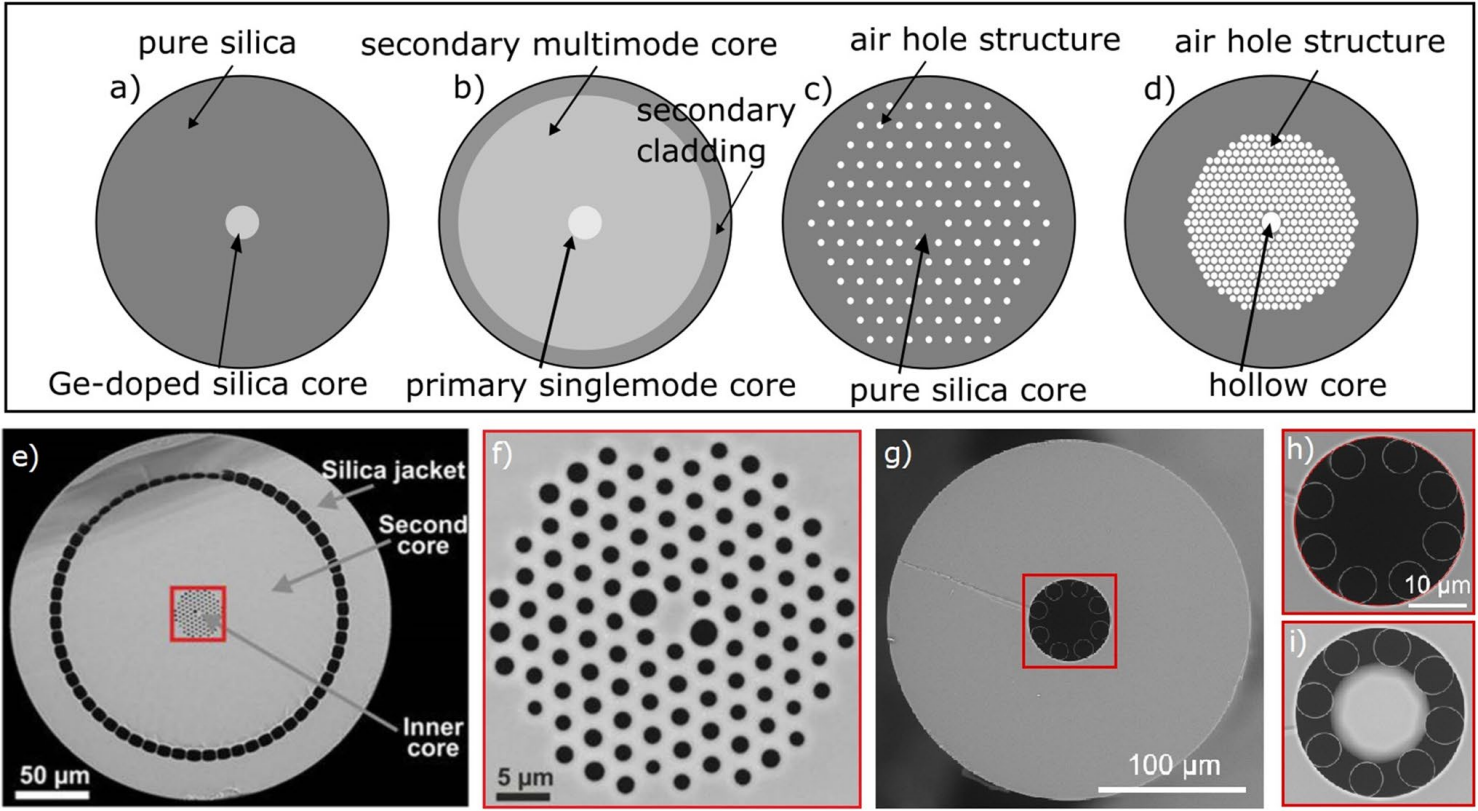
Different fiber types. **a** Doped single-mode and multimode fibers (differ in the core diameter); **b** doped double clad fiber; **c** total internal reflection (TIR) air-silica micro-structured fibers; **d** hollow core photonic bandgap fibers (HC-PBF). **e,f** Double clad photonic crystal fiber (DC-PCF) [16]: large secondary core (**e**) is used for fluorescence signal collection, while thin inner core (**f**) ensures single-mode propagation of excitation pulses. **g,h,i** Negative curvature double clad fiber: **g** cross-section of primary and secondary cores (the outer cladding made of a low index polymer was removed before imaging), **h** close-up of primary hollow single-mode core, **i** primary core with fused silica micro bead [36].

Conventional single-mode fibers (SMF) consist of a small (usually Ge-doped silica) core (3–7 µm diameter for NIR wavelengths), surrounded by a medium of a lower optical density (usually pure silica) called cladding. Light propagation in them is based on total internal reflection (TIR)—the light is reflected from core-cladding boundary, theoretically without any losses. Due to their small core diameter, SMF are well suitable for delivery of the excitation pulses. However, SMF usually have low numerical aperture (NA), meaning that the range of possible angles for the entering light to be correctly transmitted through the fiber is very limited. While that is not problematic for the alignable excitation, the direction of the fluorescence signal is random. Therefore, to collect as much emission light as possible, high sample side NA and wide collection surface is preferred. In optical confocal endoscopy, this limitation is balanced by the fact that a small core size can function as a pinhole, effectively blocking the light from out of focal planes. However, in two-photon microscopy, the origin of all scattered light is the focal point. Collecting as much fluorescence as possible increases the signal strength without limiting 3D capability and resolution. Even though they were used in some earlier multiphoton endoscopy studies [37], limited fluorescence collection possibilities make SMF fibers not the optimal choice for multiphoton imaging.

Ge-doped multimode fibers (MMF) have a larger core (10 to several 100 µm diameter) than SMF and consequently a higher NA. While well suited for fluorescence signal collection in two-photon microscopy, they cannot effectively deliver the excitation pulses [38]. Differently from SMF, the wide core and high NA of MMF allow the light pattern to contain a few stable intensity peaks, propagating side by side (transverse modes). The optical distances that the modes travel during such propagation might differ untraceably, e.g., due to fiber bending. Although for longer pulses this effect is negligible, femtosecond pulses as used in two-photon microscopy can be prolonged several times due to this intermodal dispersion, greatly reducing their intensity. Therefore, MMF cannot be used for multiphoton excitation.

#### Conventional Double Clad Fibers (DCF, Fig. 3b)

Naturally, the question arises if SMF and MMF can be combined into one. This task is accomplished in the double clad fibers (DCF). Ge-doped DCF have a highly doped narrow single-mode core (3–7 µm diameter for NIR wavelengths) for excitation pulses, a less doped wide first cladding capable of multimode wave guidance (i.e., 100-µm diameter, or more), and finally a large outer cladding. However, the need for high doping results in increased power losses due to scattering and possible small refractive index variations. Additionally, such fibers often show auto-fluorescence, adding noise to acquired images. However, this type of fiber is an acceptable candidate for multiphoton endoscopy [14, 24]. In contrast to Ge-doping the core to increase refractive index, the cladding might be F-doped to decrease refractive index, creating similar waveguiding conditions, but avoiding issues related with core impurity mentioned above. Such DCF fibers are also successfully applied in multiphoton endoscopy [39].

#### Air-Silica Micro-structured Fibers (Fig. 3c and d)

Alternatives for doped fibers (SMF, MMF, DCF) are photonic crystal fibers (PCF), a collective name for non-doped air-silica micro-structured fibers. Depending on the exact kind of micro-structure, their working principles can be divided into three groups:

- Total internal reflection (TIR) or high-index guiding fibers (Fig. 3c)
  The working principle of TIR micro-structured fibers is entirely the same as that of the Ge-doped DCF described in B. However, instead of doping the core, air holes are inserted into the cladding, reducing effective refractive index. In double clad PCF (DC-PCF, Fig. 3e and f), multiple concentric cores are constructed with separate layers of cavity micro-structures for each core. The inner core (usually 3–8 µm in diameter) is dedicated to work as a single-mode guidance for excitation pulses. In case of a solid inner core, excitation pulses experience severe nonlinear effects, which are addressed with various compensation mechanisms, discussed in the pre-compensation paragraph. The secondary core is arranged to collect the fluorescent signal and therefore has a much larger diameter and multimode guidance. In commercially available DC-PCFs, secondary cores of 90–110-µm diameter are most common, whereas some studies use specifically designed 3.5-µm inner and up to 188-µm diameter outer cores [16] for increased excitation pulse localization and better collection of the fluorescent signal.
- Photonic bandgap fibers (PBF, Fig. 3d)
  In this kind of fibers, the photonic bandgap effect is utilized for light guidance. The air hole lattice of well-controlled dimensions creates a specific photonic bandgap inside the cladding. This means that a specific band of the spectrum is restricted of entering the lattice, keeping it guided in the core. In these fibers, the refractive index of the core can be lower than that of the cladding, possibly also hollow (hollow-core photonic bandgap fibers, HC-PBF), leading to very low nonlinearity and GVD. Such fibers allow endoscopic systems without any need for pre-compensation. The drawback of HC-PBF, however, is that guidance of light is usually possible only in a relatively narrow wavelength region (100–200 nm width). Furthermore, the photonic crystal cladding must be wide to reach bandgap effect, while it cannot work as a secondary core; thus, they are only used in two-fiber systems, where minimization of the probe is not crucial [40, 41].
- Negative curvature fibers (Fig. 3g, h, and i)
  With recent advancement in fiber production, complex hollow core double clad fibers became possible (HC-DCF). It was shown that hollow core fiber featuring Kagome photonic bandgap lattice [42] or negative curvature (NC) [36] structures can effectively transport wide bandwidth excitation pulses (700–1100 nm). Specifically, the NC fibers require considerably smaller cladding structure to keep effective wave guidance in the primary core, leaving a wide area for the secondary core.

One major limitation of all hollow-core fibers described in (b) and (c) is the relatively big core size (tens of micrometers), limiting achievable resolution and two-photon excitation efficiency. It was shown previously that forming the fiber tip into the lens might increase the resolution in SMF and DCF [43]. For HCF, fusing a small (e.g., 42-µm diameter) silica bead at the distal end of the fiber (Fig. 3i) proved to be an effective solution [36, 42]. This microsphere acts as a ball lens, increasing NA tenfold and focusing laser beam to a 1.45 µm width spot (FWHM), which can be further re-imaged by the objective of the endoscope. Even though the complexity of manufacturing such fiber and correctly splicing the microsphere requires high expertise, it provides the system exceptional properties—nonlinear effects in the core of such fiber are negligible, allowing the system to act without complex compensation mechanism, while maintaining competitive resolution. It is worth mentioning that recently fiber tip engineering was also applied to conventional DCF, gluing a micro-GRIN lens to create cascaded NA amplification [44].

Conclusively, specific requirements for short excitation pulse and good VIS collection have led to increased interest in the different double clad fiber types. With technological advancement, new types of fibers are being developed, specifically designed for nonlinear endoscopic applications. Most importantly, the technology is already sufficiently effective to transport required signals to and from endoscopic probes for two-photon imaging.

### Choice of Focusing Systems

To fit in endoscopic probe, focusing optics of small diameter are required. However, the smaller optics means lower numerical aperture (NA), leading to reduced signal collection. Furthermore, conventional single lenses have slightly different focal length for different wavelengths, so-called chromatic aberration. Since in two-photon microscopy the wavelength of the fluorescence light is roughly two times shorter than that needed for excitation, wide-band achromatic compound lenses have to be used so that two-photon induced fluorescence light can be efficiently collected. Achromatic lenses consist of multiple different materials combined in such a way that the broad band of wavelengths is focused at the same distance. Some studies report objectives built of achromatic lens systems [16, 20, 45, 46]. In addition to being not easy to assemble, such systems still usually have a lower sample side NA. Earlier studies described miniature objectives composed of three achromatic doublets [16]. Recent setups include more sophisticated four doublets compositions [42], with optimized chromatic aberration correction and NA. There are also solutions for high NA (i.e., 1.0) on the sample side with great achromaticity; however, it is a tradeoff for the working distance, limiting the reachable depth in the tissue [47].

In contrast, gradient index (GRIN) lenses have arbitrary dimensions as the refraction in them is achieved by chemical composition. GRIN lenses make the assembling task easier because most usually they are produced in cylindrical form with flat entrance and exit surfaces. Furthermore, they provide significantly higher NA on sample side. However, they severely suffer from chromatic and off-axis aberrations and thus were generally acknowledged to be inferior to the achromatic lens systems [48, 20]. Recently, compound GRIN objectives with integrated optics for correcting chromatic aberrations were introduced, making them an efficient choice for multiphoton endoscopic systems [39, 49, 50]. Also, the reflective coating was reported to significantly increase collection efficiency [51]. Nonetheless, there is still room for improvement, as current two-photon microscopy-optimized GRIN lenses have very small working distance and field of view (FOV), limiting usability of such endoscopes for deeper lying structures.

### Method of Scanning and Scanning Patterns

While solutions for scanning systems are numerous and various, we are introducing them only shortly, as more extensive overviews in this particular topic are already available [52–54].

There are two different scanning systems successfully implemented inside endoscopic probes: microelectromechanical mirror systems (MEMS) and piezoelectric positioning systems (actuators).

**Fig. 4.**
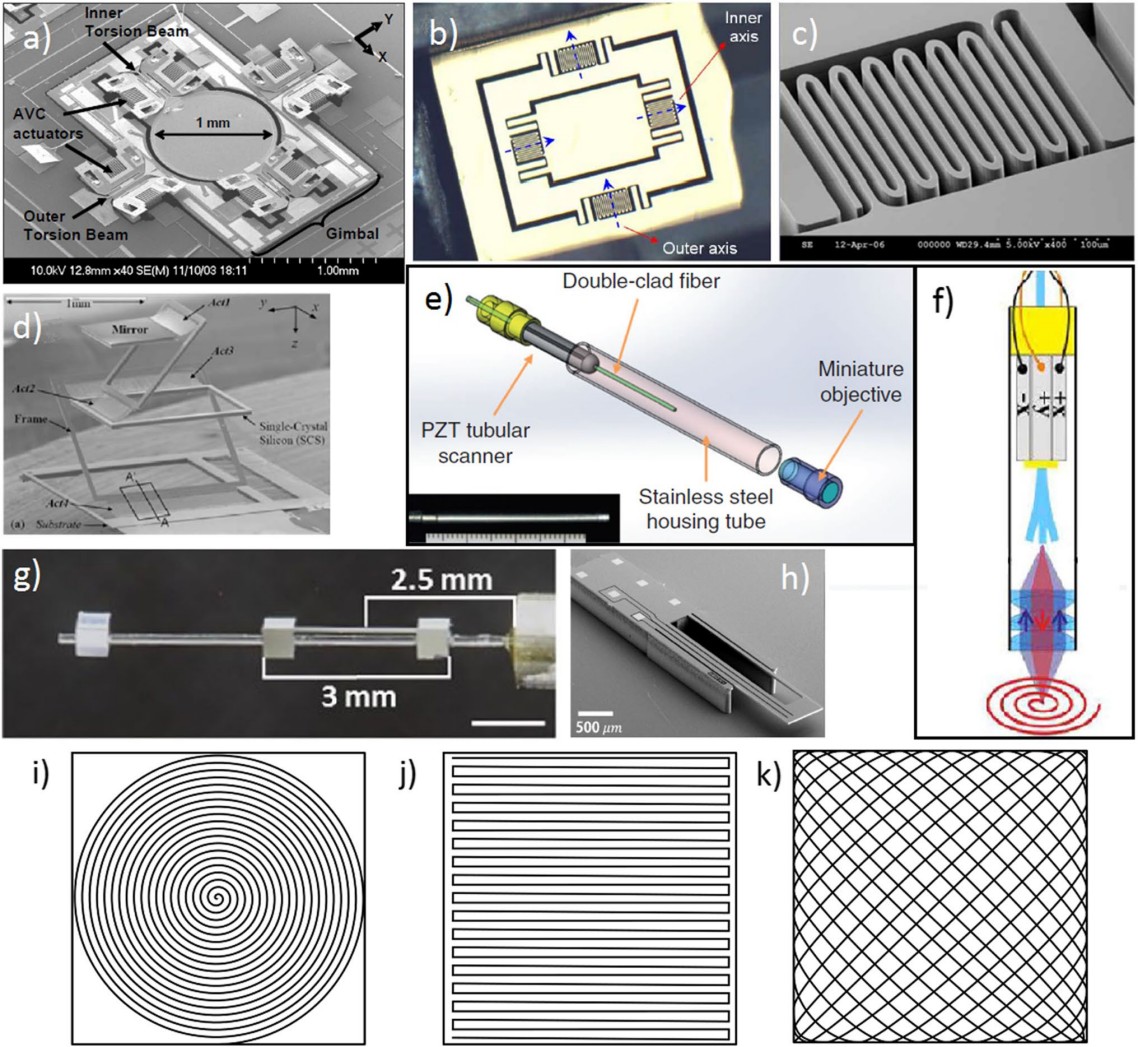
Different scanning systems and patterns. **a** Electrostatic MEMS scanning system [56]; **b,c** electromagnetic MEMS mirror scanner and close-up of a supporting flexure [58]; **d** electrothermal MEMS scanner[19]; **e,f** piezoelectric (PZT) fiber cantilever actuation systems [16, 39]; **g** PZT fiber cantilever scanning system with separated resonant frequencies for Lissajous scanning [13]; **h** thermoelectrically driven fiber cantilever scanner, capable of Lissajous scanning [59]; **i** spiral scanning pattern, common for piezo-based fiber scanners; **j** raster scanning pattern, commonly used in bench top microscopes; **k** Lissajous scanning pattern, common for MEMS-based scanners.

The scanning MEMS mirrors are operated using electrostatic, electromagnetic, or electrothermal control. Systems based on electrostatic control (Fig. 4a) allow fast scanning speed (around 5 fps), but have a low range of tilting angle, consequently limiting FOV and requiring high operational voltages [55–57]. Electromagnetic positioning (Fig. 4b, c) results in increased scanning angles under a low operational voltage, but the larger scanner operates at much lower speeds (i.e., 0.1 fps) [58]. Finally, the electrothermal control has been reported to scan 1 frame per second (Fig. 4d) [22]. The downside of MEMS systems is their complex miniaturization limited by chip size.

In the approach of piezoelectric actuators, the tip of the endoscopic fiber is scanned, which has a longer loose end part working as a cantilever [60, 61] (Fig. 4e, f, g). The length of this cantilever determines the resonant frequency of the system at which a high scanning amplitude with a low operating voltage can be reached. Fiber scanning systems reach up to 5 fps scanning rate for 512 × 512 pixel image [40] or 12 fps for 250 × 250 pixel image [16]. While most of the studies describe piezoelectric actuation of the fiber tip, there are reports of different fiber tip scanning methods, e.g., an electrothermally controlled 1.65-mm diameter scanner (Fig. 4h) [59].

Both scanning approaches—MEMS mirrors and piezo actuation—have experienced incredible improvement over the last decade. It is likely that both systems will keep improving further, as the demand for even faster scanning systems is very high. Currently achievable results are quite similar, making choice of the system dependent on manufacturing complexity. Most importantly, current state of the art of the micro-scanners already makes endoscopic TPLSM systems fast enough to be used in biological research.

Closely connected to the scanning method, three different scanning patterns can be applied in general. Piezo-based scanners most often use the outgoing spiral pattern (Fig. 4i). After scanning the full spiral frame, the scanner must return to the zero position for the next frame. The main disadvantage of such scanning pattern is that the linear speed of the fiber tip is not constant, resulting in higher scanning density in the middle of FOV. Special dynamic sampling mechanisms are being developed [21] to solve this issue. In addition, the phase difference required for correct circular spiral is very sensitive to assembly misalignment, resulting in the need for thorough testing, adjustment, and calibration [20]. Some studies have reported scanning in a raster pattern (Fig. 4j), requiring one axis to be scanned at non-resonant frequencies [25]. Such scanning pattern is similar to bench top microscopes. However, such operation requires very high operational voltages (i.e., 200 V_pp_) to achieve acceptable field of view. Recently, Lissajous scanning pattern (Fig. 4k) has become increasingly popular [13, 59, 62, 63]. This scanning pattern is more complex compared to raster or spiral patterns, and the frame rate and the resolution are more difficult to determine. Nevertheless, most MEMS scanners are typically working in such pattern in order to secure mechanical stability. Compared to spiral scanning, Lissajous scanning shows more uniform illumination over FOV, with more density in the peripheral area [64]. Therefore, some studies implement Lissajous scanning in fiber scanning systems, even when such implementation requires additional technical effort [63] (Fig. 4g, h). Hwang et al. reported Lissajous scanning systems reaching 10 frames per second rate for 256 × 256-pixel image [62].

### A New Possibility: Fiber Bundle Endoscopic Systems

It should be noted that a different approach to confocal endoscopy has been proposed very early, using a bundle of closely packed optical waveguides (fibers of large diameter with high number of independent cores arranged in a dense pattern). The device acts as an image guide, each optical core representing one pixel of the image. While image pixelation can be removed with computational techniques [65], resolution of such systems is determined by the density of optical cores. In these assemblies, bulky bench top microscopy scanners are used in the proximal end of the endoscopic fiber, reducing the complexity and dimensions of the endoscopic probe. In addition, sterilization of the endoscope is much easier, which is highly appreciated in clinical applications. Due to advances in fiber production, a few recent studies report interesting improvements in multicore fiber based nonlinear endoscopic systems [66–68]. Good resolution is achieved using very dense fiber bundles [69], or combining them with fiber scanning [70]. These improvements might make fiber bundle-based systems competitive to single fiber systems in the future. At the current state of the art, however, such systems are inferior due to poorer pre-compensation capabilities and lack of homogeneity [71].

## Applications

**Fig. 5.**
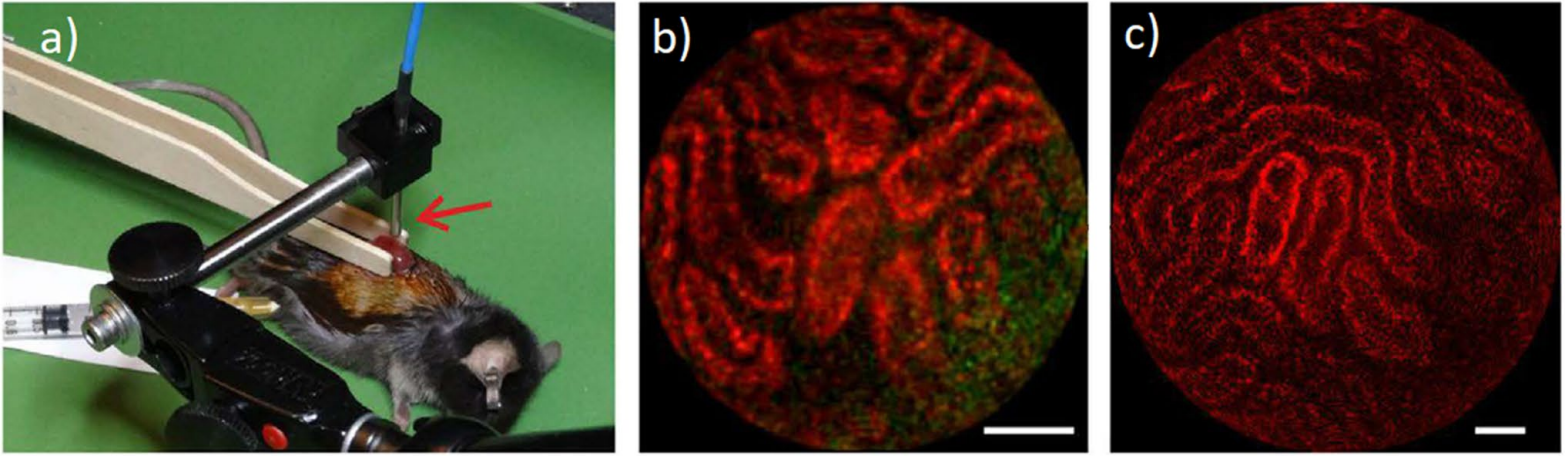
Label free *in vivo* imaging of mouse kidney. **a** Anesthetized mouse with one kidney being elevated from the body and clamped between two tongue depressors, beneath the 2.2 mm TPME probe (red arrow); **b** SHG (in green) and TPEF (in red) raw image of respectively the collagen of the capsule and the intracellular flavins of epithelial cells of the kidney tubules, **c** same as in (**b**) but with a larger FOV. Scale bars = 50 μm [16].

Multiphoton endoscopy has the potential to be useful in many different biomedical applications. However, to date, most of the biological experiments are conducted only for system validation or comparison of endoscopic capabilities with those of bench top microscopic systems. Examples of this are *in vivo* endoscopy for deep brain imaging in mice, colorectal tissue and cancer cell assessment, and rat kidney or liver tissues. Even though there are some advanced and complicated studies conducted (i.e., mouse kidney imaging *in vivo* shown in Fig. 5), very few of them really use the benefits of endoscopic capabilities in biological research, in the sense of putting a biomedical question at the forefront and use fiber-based TPLSE imaging as the only-possible tool to answer that question.

### Multiphoton Endoscopy in Deep Brain Imaging

For deep brain imaging in mice, lightweight endoscopic probes were developed [34, 41, 72–74]. These devices are in the range of tens of millimeters in dimensions and are designed not to impede free behavior. Most of them use separate fibers for excitation and fluorescence signal collection. With this application, 0.64-µm lateral and 3.35-µm axial resolution with 40 Hz frame rate 256 × 256 pixels scanning were achieved [41]. Although results of this system seem highly competitive, it must be noted that the requirements for small animal brain imaging are relatively low: (1) most of other *in vivo* applications require higher degree of probe miniaturization; (2) brain imaging is possible with longer IR wavelengths applied to a genetically modified animal model with bright intracellular fluorescent protein. Longer IR excitation pulses are easier delivered via optical fibers, as they are less susceptible to dispersion, allowing compensation-free fiber laser-based systems [75, 76], or three photon systems [77]. (3) Separate fibers for excitation and fluorescence are acceptable in this application, where brain imaging is done by creating a gap in skulls of the mice. In other internal organs a single fiber approach is required. One exception worth mentioning separately is reported by Guan et al., where the endoscopic probe used for brain imaging in freely behaving mice is 2.8 mm in diameter, which could be potentially used for other organs as well [74]. While the reported system was not exceptional in frame rate (1.5 Hz) or FOV (120 µm), it featured two laser excitation lines (830 nm and 1050 nm) simultaneously, opening new avenues in exploring brain function in freely behaving animals.

### Multiphoton Endoscopy in Colon Imaging

**Fig. 6.**
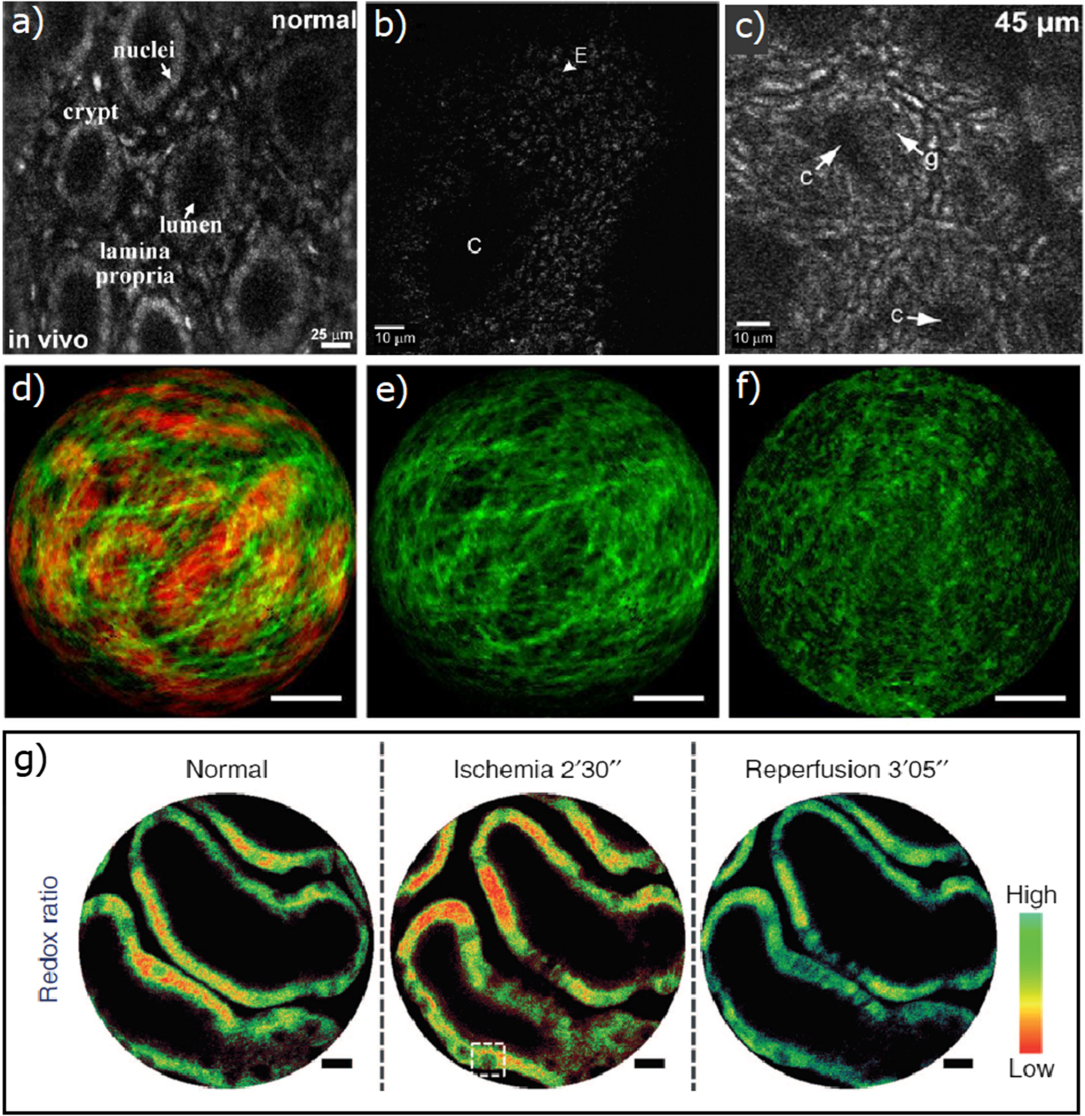
TPLSM endoscopic images of internal organs. **a** Single frame from video of normal colonic mucosa collected *in vivo* [78]; **b** image 20 to 30 µm below the surface of the interior colon showing a cross-sectional view of a crypt [79]; **c** image 45 µm below the surface with crypt (C) and goblet cells (g) [25]. **d,e,f** Label-free endoscopic images of kidney capsule in a mouse: **d** TPEF (in red) and SHG (in green) overlay of fibrotic kidney capsule; **e,f** comparison of collagen structures in fibrotic and healthy kidney [16]. Scale bars = 50 µm. **g** Redox ratio measurement in mouse kidney in normal conditions, during induced ischemia and reperfusion. Decreased redox ratio is visible in ischemia state [39]. Scale bars = 10 µm.

For colon imaging in mice and rats *in vivo*, smaller endoscopic probes are required. Duan et al. report repetitive imaging of colon in live mice three days apart for up to five times without any physical signs of trauma, and obtaining images comparable with histology [78]. This study was able to identify individual cell nuclei in crypt structures and in lamina propria (Fig. 6a). Their setup was based on a hand-held probe with MEMS scanner, capable of imaging 5 frames per second (400 × 400 pixels). In the 3.4-mm diameter distal end of the probe, a four achromatic lens optical system was designed, allowing 2-µm-lateral and 9-µm-axial resolution. Similarly to the previous report, the relatively bulky hand-held part hinders the compatibility of such probe for other applications. Brown et al. and Rivera et al. report autofluorescence images of colon tissues with enterocyte cells and some subcellular details visible (Fig. 6b [79], 6c [25]). Their system featured a piezoelectric actuators-based fiber scanner and GRIN lens objective encapsulated in a 3-mm diameter and 4-cm length stainless steel tube. The system was capable to image 4.1 frames per second (512 × 512 pixels), and 0.8-µm-lateral and 10-µm-axial resolutions were achieved. Although this system exhibits a reasonable frame rate and sufficiently small dimensions for internal applications, the raster scanning system requires high operating voltage, leading to safety issues.

### Multiphoton Endoscopy in Kidney Imaging

A few studies report images of label-free kidney in mice or rats, *in vivo* [16, 39, 46, 79] or ex vivo [21]. Ducourthial et al. obtained the second harmonic images of fibrotic and healthy kidney capsules in a mouse [16] (Fig. 6d, e, and f). To reduce motion artefacts (respirational or heartbeat), the organ was held away from the body using two tongue depressors. Using an excitation of 810 nm, they observed the renal capsule made of a collagen type I-layer overlying tubules of kidney cells in SHG and TPEF channels, respectively. The results were confirmed with high SN bench top TPLSM images obtained post mortem. The TPLSME system presented an advanced pre-compensation mechanism, reducing the duration of excitation pulses to sub-40 fs and consequently reaching high two-photon absorption efficiency. The endoscopic probe, consisting of piezoelectric fiber scanner and 3-achromat lens objective, was encapsulated in a 2.2-mm-diameter and 37-mm-length biocompatible steel tube. The 0.8-µm transverse and 12-µm axial resolutions were achieved. The scanning system allowed imaging 8 frames per second (62,500 pixels in spiral pattern).

### Multiphoton Endoscopy in Determining the Metabolic State

Liang et al. have implemented a system capable of measuring nicotinamide adenine dinucleotide (NADH) and oxidized flavin adenine dinucleotide (FAD) [39]. Their combined imaging allows the evaluation of metabolic status through two-photon excited autofluorescence. In their experiment, the left renal artery and the vein of an anesthetized mouse were clamped and then released to allow reperfusion, while changes in the redox ratio of renal cortical tubules were monitored (Fig. 6g). Redox ratio, defined as FAD/(FAD + NADH), decreased markedly in induced ischemia state, whereas NADH intensity increased. After allowing reperfusion, both changes were reversed. Significant differences in the redox ratio were found comparing ischemia state with normal or reperfusion state. The endoscopic probe contained a piezoelectric scanner and GRIN lens objective, inside of a 2.1-mm-diameter and 35-mm-length housing tube. The scanner was capable of 2.6 frames per second (512 spirals frame), and the objective allowed a 0.7-µm-lateral and 6.5-µm-axial resolution.

### Fluorescence Lifetime Imaging

Finally, some groups report fluorescence lifetime imaging (FLIM) microscopy in a fiber-based two-photon endoscope [35, 80, 81]. FLIM systems are capable of registering relaxation time of fluorophores, essentially adding one more dimension to the acquired data. It adds possibility to distinguish different substances fluorescing the same wavelength but with different relaxation times. FLIM also opens avenues to distinguish different states of a single fluorophore depending on various physical and chemical environments.

## Future Potential of Two-Photon Endoscopy

In this paragraph, we discuss the avenues opened by the availability of a user-friendly two-photon endoscopic system. Such a system allows to do rapid (few frames per second), high-resolution (cellular to subcellular), deep-tissue imaging (up to hundreds of micrometers), over reasonable fields of view (a few hundred micrometers width). While bulky standard bench top systems do allow (and are used for) deep-tissue analysis of fresh patient tissues [82], their application in biomedical and clinical research is significantly hampered due to the fact that imaging is restricted to a highly specialized microscopy lab. Instead, with a needle-like objective of mm-diameter, connected to a movable supporting system, the microscope arrives at the sample.

A movable two-photon system has already been realized, tested, and clinically applied [83]. Using the pivotal DermaInspect, potential skin tumors are investigated in presence of the patient. Subsequent two-photon, SHG, and CARS characterizations allow rapid and immediate diagnosis, instead of having to wait a week for the results of pathology. This system, however, still makes use of a large objective that is placed on the skin.

A thin needle-like objective, however, would open many additional avenues, an immediate advantage being that imaging deeper in tissue is made possible using minimally invasive and damaging protocols. Simply by gently pushing the needle into the soft tissue, so-far unreachable regions can be imaged in the intact situation.

Also, monitoring and adapting the development of tissue engineering processes under sterile conditions comes within reach. Currently, to characterize the production process, tissue-engineered samples have to be sacrificed for pathological procedures at various time points. One can only hope that these sacrificed tissues are good representative for the tissues that do reach the end stage of production. Similarly, the end-produced tissues cannot be tested and have to be trusted to be optimal for implantation. With an endoscopic system, bioimplants (e.g., heart valves, skin, and organoids) can be grown in a chamber with an integrated endoscopic system. At pre-set and fully-automated time points, each individual tissue can be imaged, without need to sacrifice or disturb the sterility of growing process. If needed, the growing protocol can be adapted to that individual tissue. Consequently, an optimal implantable tissue is the result.

Besides the advantages during ex vivo tissue imaging, two-photon endoscopy also has significant consequences for *in vivo* imaging. Indeed, deep-lying organs or tissues, unreachable by bulky objectives, can be visualized using the needle connected to a flexible fiber. Examples of these from our own research fields are as follows:

1. Imaging of the 3D structure and pathology in *in vivo* animal models, such as atherosclerotic lesions and damaged endothelial wall in carotid arteries, malformations in heart and heart valves, micro-environment within tumors, and beta-amyloid lesions in the brain.
2. Imaging of (internal) organ functionality in *in vivo* animal models, e.g., rolling of white blood cells in atherosclerotic carotids, fluctuations in endothelial glycocalyx in the vessel wall, and formation of angiogenic vasculature in tumors. All these aspects can be quantified in 3D.
3. In-patient imaging during, e.g., surgery. To-date, microscopic brightfield imaging of the glycocalyx thickness fluctuations in superficial microcirculation during brain surgery of epilepsy patients [84–87] is already applied. The endoscopic two-photon system would allow multi-faceted 3D imaging of structure and function deeper in the brain. As another example, macroscopic widefield (NIR) fluorescence imaging is currently used for the determination of the dissection region of tumors (image-guided tumor dissection [85–88]). Two-photon endoscopy would allow deeper imaging with more details, thus giving an unprecedented look into the (potentially) diseased tissue.
4. Imaging and diagnosis of symptomatic patients using so-called optical biopsies at the bedside. The needle is gently moved into the tissue under investigation and imaged without further damage. Examplatory, the imaging of placental structure and function in suspected pre-eclampsia patients. Another example could be early detection of deeper-lying tumors, i.e., in intestines or bladder. To date, one has to wait for the onset of symptoms of the disease. At that already late stage, intervention often is barely possible, and the consequences for the patient are unavoidable, sometimes even resulting in death. Thus, earlier diagnosis and potential determination of the underlying processes using endoscopic imaging could increase treatment options significantly.

Of course, to realize all these examples with a new endoscopic system, many issues have to be addressed. Firstly, imaging data obtained using two-photon endoscopes have to be related to pathology as a current golden standard. This relation is already being addressed, i.e., for skin tumors [83] and breast tumors [82]. Another issue is the safety of femtosecond-pulsed lasers, which has to be characterized for each separate tissue under investigation. Thirdly, especially for patient applications, the development of clinical fluorescent markers is essential. Nevertheless, in spite of all these essential steps and developments, we are convinced that two-photon endoscopy holds great promise for future biomedical and clinical application.

## Conclusion

Technological advances and innovative engineering led to miniaturization of probes for fiber-based multiphoton laser scanning micro-endoscopy. The current state of the art allows very interesting and beneficial biological researches in yet unexplored areas *in vivo*, which have never been reached by sub-micrometer resolution microscopy. Most importantly, the current techniques open access of TPLSME to be used in areas not covered by other instruments.

There are wide possibilities for the technology to advance and improve further, and the scientists focusing in this topic are bringing their systems closer to their goal every day—to integrate this sophisticated system into user friendly environment and make non-photonics specialists able to take advantage of it. We strongly believe that such goal will be reached in the nearest future. However, to fully employ the benefits of such imaging technique, daring biological experiments and ideas must be proposed, which could not be conducted before. Multiphoton microscopy is still mostly rendered as ex vivo or *in vitro* 3D alternative of histology, but with innovative endoscopic applications, it opens up the possibility of providing new groundbreaking, i.e., histological, insights *in vivo*. In our opinion, time has come to explore the real application of fiber-based endoscopic MPLSM to answer biological questions.
